# Precuneus Activity during Retrieval Is Positively Associated with Amyloid Burden in Cognitively Normal Older *APOE*4 Carriers

**DOI:** 10.1523/JNEUROSCI.1408-24.2024

**Published:** 2025-01-09

**Authors:** Larissa Fischer, Eóin N. Molloy, Alexa Pichet Binette, Niklas Vockert, Jonas Marquardt, Andrea Pacha Pilar, Michael C. Kreissl, Jordana Remz, Jennifer Tremblay-Mercier, Judes Poirier, Maria Natasha Rajah, Sylvia Villeneuve, PREVENT-AD Research Group, Anne Maass

**Affiliations:** ^1^German Center for Neurodegenerative Diseases (DZNE), Magdeburg 39120, Germany; ^2^Division of Nuclear Medicine, Department of Radiology & Nuclear Medicine, Faculty of Medicine, Otto von Guericke University Magdeburg, Magdeburg 39120, Germany; ^3^Clinical Memory Research, Faculty of Medicine, Lund University, Lund 223 62, Sweden; ^4^Douglas Mental Health University Institute Research Centre, McGill University, Montréal H4H 1R3, Canada; ^5^Institute for Biology, Otto von Guericke University Magdeburg, Magdeburg 39120, Germany; ^6^Department of Psychiatry, McGill University, Montréal H3A 1A1, Canada; ^7^Department of Psychology, Toronto Metropolitan University, Toronto M5S 1A1, Canada

**Keywords:** amyloid, *APOE*4, episodic memory retrieval, functional hyperactivity, multimodal neuroimaging, precuneus

## Abstract

The precuneus is a site of early amyloid-beta (Aβ) accumulation. Previous cross-sectional studies reported increased precuneus fMRI activity in older adults with mild cognitive deficits or elevated Aβ. However, longitudinal studies in early Alzheimer's disease (AD) are lacking and the relationship to the Apolipoprotein-E (*APOE*) genotype is unclear. Investigating the PREVENT-AD dataset, we assessed how baseline and longitudinal precuneus activity during successful memory retrieval relates to future Aβ and tau burden and change in memory performance. We further studied the moderation by *APOE*4 genotype. We included 165 older adults (age, 62.8 ± 4.4 years; 113 female; 66 *APOE*4 carriers) who were cognitively normal at baseline with a family history of AD. All participants performed task-fMRI at baseline and underwent ^18^F-flortaucipir-PET and ^18^F-NAV4694-Aβ-PET on average 5 years later. We found that higher baseline activity and greater longitudinal increase in precuneus activity were associated with higher Aβ burden in *APOE*4 carriers but not noncarriers. We observed no effects of precuneus activity on tau burden. Finally, *APOE*4 noncarriers with low baseline precuneus activity exhibited better longitudinal performance in an independent memory test compared with (1) noncarriers with higher baseline activity and (2) *APOE*4 carriers. Our findings suggest that higher task-related precuneus activity during memory retrieval at baseline and over time are associated with greater Aβ burden in cognitively normal *APOE*4 carriers. Our results further indicate that the absence of “hyperactivation” and the absence of the *APOE*4 allele is related with better future cognitive outcomes in cognitively normal older adults at risk for AD.

## Significance Statement

The precuneus, a brain region involved in episodic memory, is a site of early amyloid-beta (Aβ) accumulation. Alterations in task-related activity occur in the precuneus with aging and with Alzheimer's disease (AD) pathology even in the absence of cognitive symptoms; however, their course and implications are not well understood. We demonstrate that higher precuneus activity at baseline and its change over time during successful memory retrieval is associated with higher Aβ burden on average 5 years after baseline in Apolipoprotein-E4 (*APOE*4) carriers. Lower precuneus baseline activation was related to better longitudinal memory performance in *APOE*4 noncarriers. Our findings provide novel longitudinal evidence that increased activity in posterior midline regions is linked to early AD pathology in dependence of *APOE*4 genotype.

## Introduction

Changes in brain activity occur across the normal life course and in early Alzheimer's disease (AD) and can be measured indirectly with functional magnetic resonance imaging (fMRI). Understanding how these changes are mechanistically linked to progression of AD offers opportunities for preventing cognitive decline (Corriveau-Lecavalier et al., 2024). The precuneus, which is part of the posteromedial cortex (PMC), is among the earliest regions affected by amyloid-beta (Aβ) pathology (Villeneuve et al., 2015; Palmqvist et al., 2017), rendering it a promising region to investigate early aberrant activity. Furthermore, the precuneus is strongly involved in episodic memory processing (Cavanna and Trimble, 2006; Elman et al., 2013; Moscovitch et al., 2016), a domain that declines in healthy aging and early AD (Hedden and Gabrieli, 2004; Rönnlund et al., 2005; McKhann et al., 2011).

Several lines of research suggest a role for precuneus dysfunction in cognitive aging and AD pathogenesis. For example, lower task-related precuneus deactivation, particularly during encoding, has been observed in older adults relative to younger adults (Lustig et al., 2003), suggesting heightened activation with age. Interestingly, this finding has been replicated in different cohorts and tasks (Miller et al., 2008; Pihlajamäki et al., 2008; Bejanin et al., 2012; Mormino et al., 2012; Fenerci et al., 2022; Kizilirmak et al., 2023). Similarly, increased precuneus activity during encoding has also been observed in older adults with subjective cognitive decline (SCD; Corriveau-Lecavalier et al., 2020; Billette et al., 2022), individuals at increased risk of developing AD dementia (Mitchell et al., 2014; Slot et al., 2019). While PMC regions strongly activate during successful memory retrieval (for review, see Kim, 2013), studies investigating how retrieval activity in these regions is altered in early AD are scarce (McDonough et al., 2020). Research focusing on aberrant hippocampal activity (Yassa et al., 2010; Leal et al., 2017; Tran et al., 2017) points to this region as a potential target for therapeutic intervention, with findings linking antiepileptic medications to reduced hippocampal activity and behavioral improvements (Bakker et al., 2012, 2015). Recently, efforts to reduce cognitive impairment by targeting aberrant precuneus activity and connectivity have been made (Koch et al., 2018; Millet et al., 2023). Several studies in unimpaired older adults reported associations between reduced precuneus deactivation during encoding and higher Aβ burden (Sperling et al., 2009; Vannini et al., 2012). Further studies have also found associations of the Apolipoprotein-E4 (*APOE*4) genotype and heightened PMC activity (Han et al., 2007; Persson et al., 2008; Pihlajamäki et al., 2010). *APOE*4 is a major risk factor for AD (Mayeux, 2003; Liu et al., 2013) and is strongly correlated with Aβ accumulation (Villemagne and Rowe, 2013; Selkoe and Hardy, 2016). Recently, it has been proposed that *APOE*4 homozygosity represents a distinct form of genetic AD, with almost all homozygotes showing AD pathology and cognitive symptoms in later life (Fortea et al., 2024). Therefore, there seems to be accumulating evidence for a role of aberrant hyperactivation of PMC regions, in addition to the well-established risk associated with *APOE*4 genotype, in the preclinical stages of AD. However, how these two factors interact to affect the spread of AD pathology remains unclear and to be empirically tested.

Here we assessed the relationship between precuneus fMRI retrieval activation at baseline and change over time, *APOE*4 genotype, cognitive changes, and AD pathology in cognitively normal adults from the longitudinal Pre-symptomatic Evaluation of Experimental or Novel Treatments for Alzheimer's Disease (PREVENT-AD) cohort. PREVENT-AD incorporates multimodal data from cognitively unimpaired older adults with a familial history of sporadic AD (Tremblay-Mercier et al., 2021). We hypothesized that (1) precuneus brain activity would be higher or increasing over time in *APOE*4 carriers compared with noncarriers, (2) increased precuneus activity at baseline and increases over time would be linked to future Aβ and tau burden, (3) higher activity or activity changes would be positively (if beneficial) or negatively (if detrimental) linked to cognitive changes, and (4) *APOE* genotype moderates associations between activity, pathology, and cognition.

## Materials and Methods

### Sample and study design

All participants were cognitively unimpaired older adults from the open science PREVENT-AD cohort study launched in 2011 (Breitner et al., 2016; Tremblay-Mercier et al., 2021). Participants had at least one parent or two siblings diagnosed with AD-like dementia, which is associated with an increased risk for developing sporadic AD (Donix et al., 2012). Participants were above 60 years of age at baseline. People aged between 55 and 59 were also included if they were <15 years away from the age of onset of symptoms of their first-affected relative. Participants had no major neurological or psychiatric illnesses at time of enrolment. Inclusion criteria comprised intact cognition based on the Montreal Cognitive Assessment (MoCA) questionnaire with a score of at least 26 of 30 points (Nasreddine et al., 2005), a Clinical Dementia Rating (CDR) Scale of 0 (Morris, 1993), or exhaustive neuropsychological evaluation. All participants included in our analyses underwent at least a baseline fMRI scan with sufficient task performance to form the contrast of interest (see below, Task-fMRI preprocessing and data preparation), meaning a corrected hit rate >0.2 and a minimum of 10 hits and 10 correct rejections. A total of 374 participants had available data of at least a baseline fMRI scan; however, 55 sessions, including all sessions from 16 participants, had to be excluded because of a corrected hit rate below 0.2. Due to a failure to reach a minimum of 10 hits and 10 correct rejections, two more sessions had to be excluded. Further criteria were available cognitive assessments using the standardized Repeatable Battery for the Assessment of Neuropsychological Status (RBANS; Randolph et al., 1998) and both an Aβ and tau PET scan at varying times (mean, 5 years; range, 0.5−10 years) post-baseline fMRI scan. This selection created a subsample of 165 participants (aged 62.8 ± 4.4 years at baseline, 15.42 ± 3.3 years of education, 52 male/113 female, 66 *APOE*4 carriers including 3 *APOE*4 homozygotes) upon which our analyses were performed (Table 1).

**Table 1.** Demographic information of the sample for *APOE*4 noncarriers and carriers

	*APOE*4 noncarrier (*N* = 99)	*APOE*4 carrier (*N* = 66)	*t* or *x*^2^	*p*
Age (years)	63.4 (4.6)	62.0 (4.1)	2.034	0.044
Education (years)	15.5 (3.3)	15.3 (3.2)	0.442	0.659
Sex (male/female)	30/69	22/44	0.057	0.81
Amyloid PET burden (SUVR)	1.21 (1.16)	1.42 (0.35)	−4.260	<0.001
Amyloid positive (N)	7	25	22.113	<0.001
Tau PET burden (SUVR)	1.04 (0.1)	1.07 (0.14)	−1.650	0.102
Time baseline to PET (years)	5.13 (2.2)	5.38 (2.3)	−0.690	0.492

Mean (standard deviation) or number (N) of participants. *t* or chi-squared test for *APOE*4 noncarrier vs *APOE*4 carrier. An amyloid positivity threshold of an SUVR value of 1.39 was provided by the PREVENT-AD Research Group. *APOE*, apolipoprotein E; SUVR, standardized uptake value ratios.

**Figure 1.**
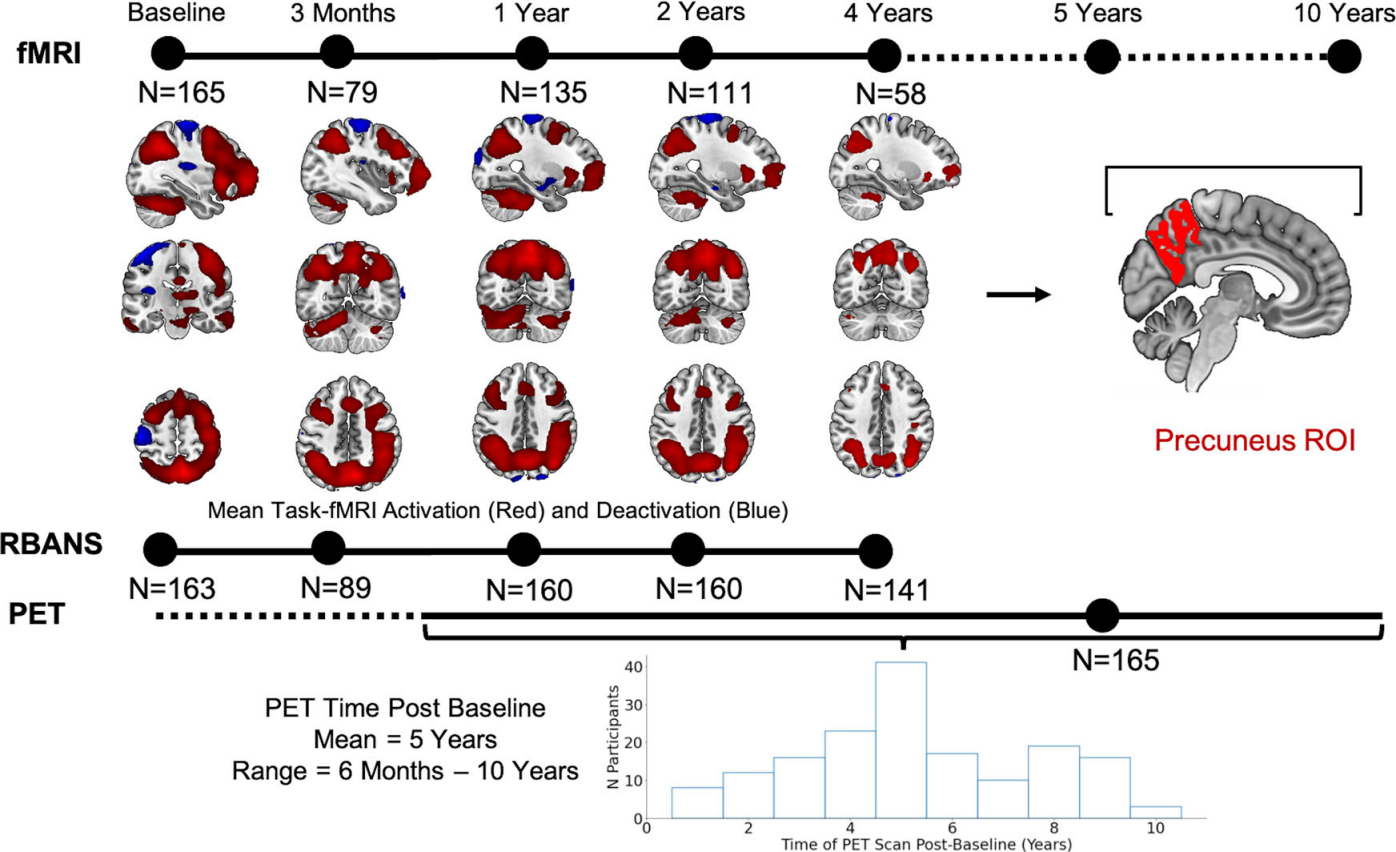
Study design: Each participant underwent one baseline fMRI session and up to four fMRI follow-up sessions with the last follow-up 4 years after baseline. Similarly, RBANS neuropsychological assessments were performed at baseline and over time. All 165 participants underwent PET scans to quantify amyloid-beta and tau pathology between 6 months and up to 10 years after the baseline fMRI scan. Group fMRI activity during successful retrieval (red scale, hits > correct rejections; inverse contrast in blue; Extended Data Fig. 1-1 for task paradigm) is depicted for each time point. FMRI results shown at *p* < 0.05 FWE-corrected at the voxel level. fMRI, functional magnetic resonance imaging; RBANS, Repeatable Battery for the Assessment of Neuropsychological Status; PET, positron emission tomography.

Follow-up fMRI scans and RBANS assessments were performed over the course of 48 months in a subset of participants. Specifically, participants underwent a 3 month (*N* = 79), 12 month (*N* = 135), 24 month (*N* = 111), and 48 month (*N* = 58) follow-up fMRI scan after baseline (Fig. 1). The 3 month follow-up was only scheduled for participants of the INTREPAD prevention substudy, described in detail in Meyer et al. (2019). All study procedures and experimental protocols were approved by the McGill University Institutional Review Board and/or the Douglas Mental Health University Institute Research Ethics Board. All participants provided written informed consent prior to each experimental procedure and were financially compensated for their time.

10.1523/JNEUROSCI.1408-24.2024.f1-1 Figure 1-1 Download Figure 1-1, TIF file.

### Task-fMRI design

FMRI data were acquired using a Siemens Tim Trio 3 tesla MRI scanner at the Cerebral Imaging Centre of the Douglas Mental Health University Institute using a Siemens standard 12 or 32-channel coil (Siemens Medical Solutions; Tremblay-Mercier et al., 2021). Scans were acquired with a TR 2,000 ms; TE 30 ms; 90° flip angle, FOV 256 × 256 mm field of view covering 32 slices, and a 4 mm isotropic voxel resolution. Participants performed an encoding and retrieval block of an object–location episodic memory task within each scan session. Details of the task fMRI methods have been previously published (Rabipour et al., 2020; Tremblay-Mercier et al., 2021). During the encoding task, participants were presented with 48 visual stimuli (colored line drawings of everyday items), presented on either the right or left side of the screen. Participants were asked to indicate on which side of the screen the stimulus was presented by pressing a button. After a 20 min interval of structural scanning, participants performed the retrieval task. They were presented with 48 old stimuli, i.e., object stimuli shown during the encoding session, and 48 new object stimuli. Specifically, participants were asked to indicate via a button press (forced-choice between four alternative answers), whether (1) “The object is FAMILIAR but you don't remember the location” (“F”); (2) “You remember the object and it was previously on the LEFT” (“L”); (3) “You remember the object and it was previously on the RIGHT” (“R”); and (4) “The object is NEW” (“N”; Extended Data Fig. 1-1). The retrieval task took ∼15 min. For the purpose of this paper, we focus on brain activity associated with successful object recognition (see also next section for details), that is, on activity differences between correctly recognized (old) objects (irrespective of location/ source memory) versus activity during correct rejection of novel objects.

### Task-fMRI preprocessing and data preparation

All data were preprocessed using MATLAB and Statistical Parametric Mapping, version 12 (SPM12; Functional Imaging Laboratory UCL, 2023). Data were realigned, slice time corrected, coregistered to an anatomical T1 image, normalized, and smoothed using a 8 mm full-width at half-maximum (FWHM) Gaussian kernel. Three-dimensional T1 anatomical data (TR 2,300 ms; TE 2.98 ms; TI 900 ms; 9° flip angle; FOV 256 × 240 × 176 mm) with a 1 mm isotropic voxel resolution were segmented for functional image normalization using the unified segmentation approach (Ashburner and Friston, 2005). Following preprocessing, we performed first-level analyses. The first-level GLM included three regressors of interest: hits (responses “F”/”L”/”R” to old object stimuli), correct rejections (response “N” to new object stimuli), and false alarms or misses (i.e., all other responses) as well as six motion regressors from the realignment process. All included participants had a corrected hit rate >0.2 and a minimum of 10 hits and 10 correct rejections, specifying a *t*-contrast, hereafter referred to as the episodic memory contrast. To specify the episodic memory contrast, we compared “hits” (previously viewed items that were correctly identified, regardless of their previously presented location on screen) with “correct rejections” (new items correctly identified as new). We chose this contrast as previous studies have consistently reported high activation of the precuneus when comparing correctly remembered items versus novel foils (Kim, 2013). Participants performed on average very well on the task with less than seven misses (mean, 27; SD = 5.42) as can be seen in Table 8. To assess precuneus brain activity associated with the episodic memory contrast, we applied a region of interest (ROI) approach using FreeSurfer (Laboratories for Computational Neuroimaging, 2023) masks (Fig. 1; labels 1,025 and 2,025 from the aparc + aseg.nii in MNI space), resliced to match functional image dimensions using the “Coregister and Reslice” command in SPM12. Using these masks, we subsequently extracted mean beta values for the bilateral precuneus during the Hits > Correct Rejections episodic memory contrast for each participant using in house MATLAB scripts.

### PET acquisition and preprocessing

PET scans were performed at the McConnell Brain Imaging Centre of the Montreal Neurological Institute (Quebec, Canada) using a brain-dedicated PET Siemens/CTI high-resolution research tomograph. Data acquisition and processing was carried out as previously described (Yakoub et al., 2023). In brief, Aβ-PET images using ^18^F-NAV4694 (NAV) were acquired 40–70 min after injection, with an injection dose of ∼6 mCi. Tau-PET images, using ^18^F-flortaucipir (FTP), were acquired 80–100 min after injection, with an injection dose of ∼10 mCi. Frames of 5 min as well as an attenuation scan were obtained. PET images were reconstructed using a 3D ordinary Poisson ordered subset expectation maximum algorithm (OP-OSEM), with 10 iterations, 16 subsets, while all images were decay and motion corrected. Scatter correction was performed using a 3D scatter estimation method. T1-weighted MRI images were parcellated into 34 bilateral ROIs based on the Desikan-Killiany atlas using FreeSurfer version 5.3. PET images were realigned, temporally averaged, and coregistered to the T1-weighted image (using the scan closest in time to PET data acquisition), then masked to remove signal from cerebrospinal fluid (CSF), and smoothed with a 6 mm Gaussian kernel. Standardized uptake value ratios (SUVRs) were computed as the ratio of tracer uptake in the ROIs versus uptake in cerebellar gray matter for Aβ-PET scans or versus inferior cerebellar gray for tau-PET. All PET data were preprocessed using a standard pipeline (https://github.com/villeneuvelab/vlpp). We focused on a ROI approach for tau, assessing bilateral entorhinal FTP SUVR, obtained by averaging the uptake ratio of both the left and right entorhinal cortices and whole-brain NAV SUVR.

### *APOE* genotyping

All participants were genotyped for *APOE* using a QIASymphony apparatus, as described previously (Tremblay-Mercier et al., 2021). If participants showed at least one copy of the *APOE*4 risk allele, they were allocated to the carrier group while those without were allocated to the noncarrier group (carriers, 66 with 22 male; noncarriers, 99 with 30 male).

### Assessment of memory performance

We focused on two measures of episodic memory, the RBANS delayed memory index score and corrected hit rate derived from the fMRI retrieval task. The RBANS delayed memory index score is a combined measure of word-list recognition and delayed figure, story, and word-list recall (Randolph et al., 1998). Corrected hit rate was specified as hits (i.e., responses “Familiar,” “Remember-Left,” or “Remember-Right” to previously shown objects) minus false alarms (responses “Familiar,” “Remember-Left,” or “Remember-Right” to novel objects) during the fMRI retrieval recognition task. We note that different versions of the RBANS were used in follow-up sessions to reduce practice effects and different object stimuli were employed at each fMRI visit. RBANS data from baseline (*N* = 163), a 3 month (*N* = 89), 12 month (*N* = 160), 24 month (*N* = 160), and 48 month (*N* = 141) follow-up were included.

### Statistical analysis

All statistical analyses were conducted using R (R Core Team, 2022), version 4.1.2, implemented within RStudio (RStudio Team, 2022), and running on macOS Monterey version 12.4. The R code used for analyses is publicly available (https://github.com/fislarissa/precuneus_retrieval_hyperactivation). For the linear models, we ensured that heteroscedasticity and multicollinearity were not present. Furthermore, we tested for a normal distribution of residuals using the Shapiro–Wilk test on the standardized residuals of each model. For linear mixed models (LMMs; Bates et al., 2015), we included a random intercept and slope. When this led to a singular fit, we restricted the model to a random intercept only. Our analyses focused on four major questions:

1. Are there differences in precuneus activity during memory retrieval at baseline or over time between *APOE*4 carriers and noncarriers?
2. Is higher precuneus activity at baseline and increase in activity over time associated with future Aβ and tau burden?
3. Is higher activity or longitudinal activity change positively (if beneficial) or negatively (if detrimental) related to cognitive changes?
4. Does *APOE* genotype moderate any association between activity and pathology or cognition (e.g., is higher activity related to more pathology only in *APOE*4 carriers)?

### Assessment of the effect of *APOE* genotype on baseline and longitudinal precuneus activation

Following extraction of precuneus-specific magnitude of brain activity at baseline, we specified a linear model to assess effects of *APOE*4 status adjusting for age at baseline, sex, years of education, and precuneus gray matter volume (GMV) at baseline, obtained from T1-weighted structural images (Baseline Precuneus Activity ∼ *APOE*4 Group *+* Age + Sex + Education + GMV). We then specified an LMM to investigate changes in activity over time, with precuneus activity as the dependent variable and time (as the scaled continuous time difference between individual sessions) as the fixed within-subject effect and random intercepts per participant, adjusting for the same variables [Precuneus Activity ∼ Time + *APOE*4 Group + Age + Sex + Education + GMV + (1|Participant)]. We then repeated the analysis including an interaction term of time by *APOE* status and as a supplementary analysis an interaction term of time by sex [Precuneus Activity ∼ Time * *APOE*4 Group + Time * Sex + Age + Education + GMV + (1|Participant)].

### Assessment of the relationship between baseline and longitudinal precuneus activity and AD pathology and its moderation by *APOE* genotype

We examined the effect of *APOE* genotype and its interaction with activity on AD pathology burden. We first tested for a difference in Aβ and tau burden between *APOE* genotype groups, adjusting for age, sex, and education.

To test whether baseline precuneus activity statistically predicted AD pathology at follow-up, we specified two linear models in which baseline precuneus activity was used as the independent and (1) whole-brain Aβ PET and (2) entorhinal tau SUVRs as the dependent variables, respectively. Age at baseline, sex, years of education, precuneus GMV at baseline, and time (in months) from the baseline fMRI scan to the respective PET scan were specified as covariates in each model (AD Pathology ∼ Baseline Precuneus Activity + Age + Sex + Education + GMV + Time Baseline fMRI to PET). Due to the non-normal distribution of Aβ and tau pathology, we applied a Box-Cox transformation to the PET data in order to achieve a closer approximation of a normal distribution.

To examine the effect of activity change on AD pathology, we next extracted the slope of the change in precuneus activity over time for each participant. The specified model for the slope extraction included precuneus activation as dependent variable, time (as the scaled continuous time difference between individual sessions) as independent variable, and a random intercept and slope per participant. Subsequently, we entered the extracted slope of activation as the predictive variable in a second set of linear regressions, assessing the effects of change in precuneus activity over time on AD pathology at follow-up. Age, sex, education, precuneus GMV at baseline, and time (in months) from the baseline fMRI scan to the respective PET scan were again included as covariates (AD pathology ∼ Precuneus Activity Slope + Age + Sex + Education + GMV + Time From Baseline fMRI to PET). To assess whether the activity slope was associated with the baseline fMRI signal, we performed a correlation analysis.

We subsequently repeated our linear regression analyses in which we tested if there was an interaction between precuneus activation and APOE4 genotype at (1) baseline (AD Pathology ∼ Baseline Precuneus Activity * *APOE* genotype + Age + Sex + Education + GMV + Time Baseline fMRI to PET) and (2) over time (slope) (AD Pathology ∼ Precuneus Activity Slope * APOE genotype + Age + Sex + Education + GMV + Time From Baseline fMRI to PET) on AD pathology at follow-up.

### Assessment of the relationship between baseline precuneus activation and baseline memory performance as well as changes in memory performance

To test for associations between baseline precuneus activation and baseline corrected hit rate (specified as hits minus false alarms) of fMRI task-performance or the delayed memory score obtained with the RBANS, we used partial correlation analyses (correcting for years of education, sex, and age). To initially test for changes in memory performance in our cohort over time, we modeled the longitudinal corrected hit rate from the task fMRI or the RBANS delayed memory index score as the dependent variable in two LMMs and time as the within-subject factor and random intercepts per participant. Age, sex, and years of education were covariates in all analyses [Memory Performance ∼ Time + Age + Sex + Education + (1|Participant)].

### Assessment of the effect of baseline precuneus activation and *APOE* genotype on longitudinal memory performance

In order to assess the interaction effects of precuneus activation and *APOE* genotype on measures of episodic memory over time, we created two LMMs in which episodic memory performance (first measured by the corrected hit rate from the task fMRI and second from the RBANS delayed memory index score) was specified as the dependent variable and precuneus activation at baseline as the independent variable. We specified session (as Sessions 1–5; to investigate session-specific differences) and *APOE* genotype as within-subject factors and random intercepts per participant. Again, this model was specified with age, sex, and education as covariates. We first investigated the interaction effects of baseline activity by session and *APOE* genotype by session [Memory Performance ∼ Baseline Precuneus Activity * Session + *APOE*4 Group * Session + Age + Sex + Education + (1|Participant)] and then a three-way interaction of baseline activity by *APOE* genotype by session (Memory Performance ∼ Baseline Precuneus Activity * *APOE*4 Group * Session + Age + Sex + Education + (1|Participant)] on memory performance. We then applied post hoc contrasts to each session for those models with significant interactions.

Finally, we investigated group effects on the performance slopes over time correcting for age, sex, and education. The specified model for the slope extraction included the respective memory performance as dependent variable, time (as the scaled continuous time difference between individual sessions) as independent variable, and a random intercept and slope per participant. We then contrasted APOE4 carriers versus noncarriers for the slope of corrected hit rate performance (Memory Performance Slope ∼ APOE4 Group + Age + Sex + Education) and APOE4 noncarriers with low baseline activation versus all other groups for the slope of RBANS delayed memory index score performance (Memory Performance Slope ∼ APOE4 and Activation Level Group + Age + Sex + Education)*.* For these post hoc comparisons regarding the RBANS slopes, we applied Tukey's test with familywise error (FWE) correction to account for multiple comparisons.

## Results

### Assessment of the effect of *APOE* genotype on baseline and longitudinal precuneus activation

**Figure 2.**
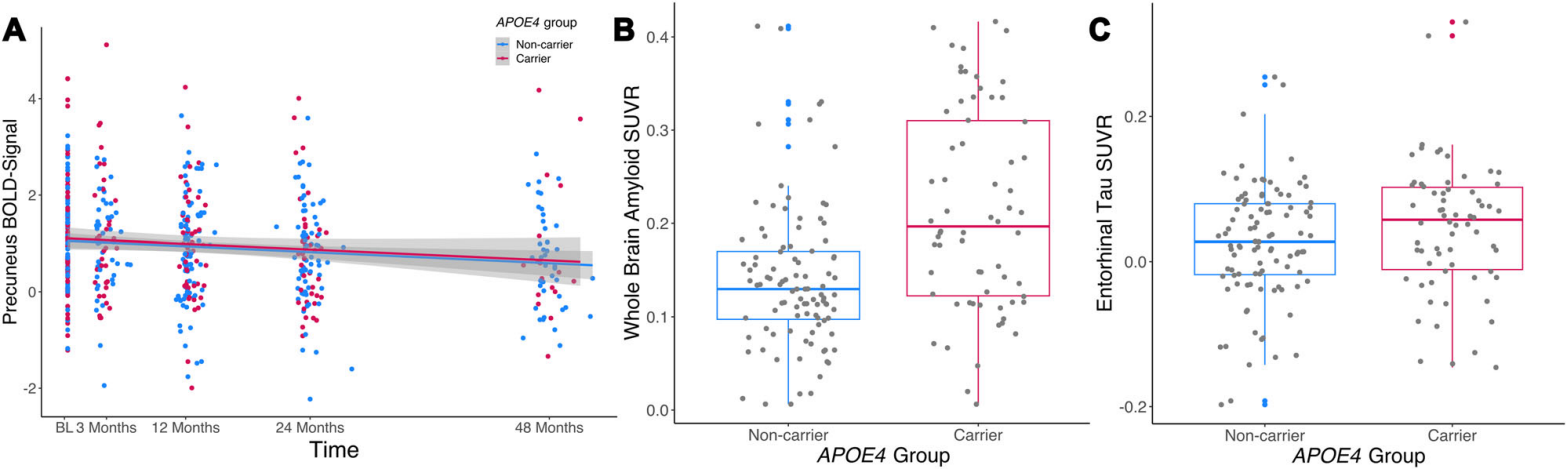
Precuneus retrieval activity over time by *APOE* genotype and Alzheimer's pathology differences between *APOE* genotype groups: ***A***, Linear mixed modeling showed a significant decrease in precuneus activity over time in the whole sample. There was no significant time by *APOE* genotype interaction, suggesting comparable changes over time in *APOE*4 carriers (red) and noncarrier (blue). Shaded areas refer to the 95% confidence interval. ***B***, Whole-brain amyloid burden was significantly higher in*APOE*4 carriers than noncarriers, when adjusting for age, sex, and years of education. ***C***, Similarly, *APOE*4 carriers also showed a marginally higher tau burden in the entorhinal cortex. BL, baseline.

Precuneus activity during memory retrieval at baseline in all (*N* = 165) participants did not differ due to *APOE*4 status (*p* > 0.05; Table 2). Regarding longitudinal changes in precuneus activity, there was a statistically significant decrease of precuneus retrieval activity over time (*β* = −0.15 [95% CI −0.22, −0.07]; *t* = −3.987; *p* < 0.001) in all participants with >1 fMRI scan (*N* = 151). There was also no significant time by *APOE* group interaction (*p* > 0.05; Table 3), with both carriers and noncarriers exhibiting similar decreases in precuneus brain activity over time (Fig. 2*A*).

**Table 2.** Linear model of effects on baseline activation

	Baseline precuneus activity							
Predictors	Estimates	Std. error	Std. beta	Standardized std. error	CI	Standardized CI	Statistic	*p*
(Intercept)	1.44	3.20	−0.15	0.11	−4.88–7.76	−0.37–0.08	0.45	0.653
*APOE*4 Group (carrier)	0.05	0.17	0.05	0.16	−0.29–0.40	−0.27–0.37	0.30	0.766
Age at baseline	0.00	0.00	0.05	0.08	−0.00–0.00	−0.11–0.22	0.65	0.515
Sex (male)	0.44	0.19	0.41	0.18	0.06–0.82	0.06–0.76	2.30	0.023
Education at baseline	0.01	0.03	0.03	0.08	−0.04–0.06	−0.13–0.18	0.35	0.725
Precuneus GMV	−1.97	3.45	−0.05	0.09	−8.79–4.84	−0.22–0.12	−0.57	0.568
Observations	165							
*R*^2^/*R*^2^ adjusted	0.056/0.026							

Precuneus activation at baseline was used as dependent variable, APOE4 status, age at baseline, sex, education, and precuneus gray matter volume were used as independent variables. Male participants showed higher activity at baseline. GMV, gray matter volume. CI, 95% confidence interval.

**Table 3.** Linear model of effects on change in precuneus activation including the interaction term of time by *APOE* genotype and time by sex

	Precuneus activity									
Predictors	Estimates	Std. error	Std. beta	Standardized std. error	CI	Standardized CI	Statistic	Std. statistic	*p*	Std. *p*
(Intercept)	−0.76	2.43	−0.12	0.08	−5.57–4.05	−0.29–0.05	−0.31	−1.39	0.754	0.167
Time	−0.00	0.00	−0.08	0.05	−0.00–0.00	−0.18–0.02	−1.64	−1.64	0.102	0.102
*APOE*4 group (carrier)	0.08	0.15	0.09	0.12	−0.22–0.38	−0.16–0.33	0.54	0.72	0.589	0.474
Age at baseline	0.00	0.00	0.08	0.06	−0.00–0.00	−0.04–0.21	1.29	1.29	0.199	0.199
Sex (male)	0.46	0.17	0.23	0.13	0.13–0.79	−0.03–0.50	2.74	1.72	0.007	0.088
Education at baseline	0.00	0.02	0.01	0.06	−0.03–0.04	−0.11–0.13	0.16	0.16	0.875	0.875
Precuneus GMV	0.49	2.63	0.01	0.06	−4.71–5.68	−0.11–0.14	0.18	0.18	0.854	0.854
Time × sex (male)	−0.00	0.00	−0.20	0.08	−0.00–−0.00	−0.35–−0.05	−2.61	−2.61	0.009	0.009
Time × *APOE*4 group (carrier)	0.00	0.00	0.01	0.08	−0.00–0.00	−0.14–0.17	0.19	0.19	0.852	0.852
Random effects										
σ^2^	0.77									
τ_00_ Subject	0.39									
ICC	0.33									
*N* Subject	151									
Observations	534									

Precuneus activation over time was used as dependent variable, time, *APOE*4 status, age at baseline, sex, education, precuneus gray matter volume, and the interaction of time by *APOE* as well as of time by sex were used as independent variables. There was only a significant time interaction with sex as shown in Extended Data Table 3-1. GMV, gray matter volume. CI, 95% confidence interval.

10.1523/JNEUROSCI.1408-24.2024.t3-1 Table 3-1 Download Table 3-1, TIF file.

### Assessment of the relationship between baseline and longitudinal precuneus activity and AD pathology and its moderation by *APOE* genotype

Regarding AD pathology burden, *APOE* carriers exhibited significantly higher whole-brain Aβ (*β* = 0.79; [95% CI 0.50, 1.09]; *t* = 5.335; *p* < 0.001; Fig. 2*B*) and entorhinal tau burden (*β* = 0.32; [95% CI 0.01, 0.63]; *t* = 2.040; *p* = 0.043; Fig. 2*C*).

**Figure 3.**
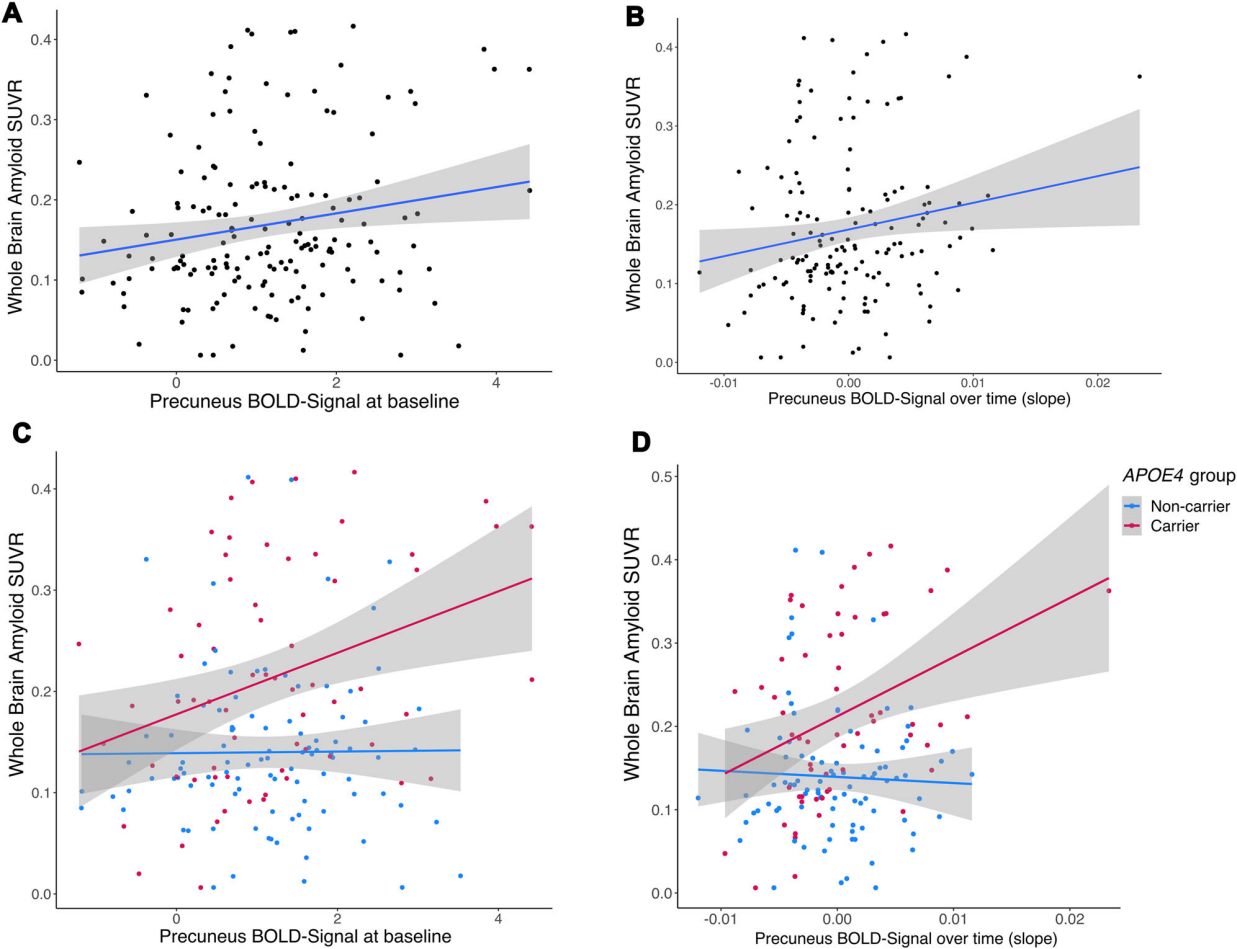
Relationship between precuneus activation and PET-assessed measures of amyloid burden. ***A***, A linear regression model showed that baseline precuneus activation was significantly related to later whole-brain amyloid (Aβ) burden. ***B***, Change in precuneus activation over time was also significantly associated with Aβ PET burden, with a steeper positive slope being associated with higher Aβ burden. ***C***, *APOE* genotype moderated the association between precuneus activation at baseline and future whole brain Aβ burden (with higher brain activation levels at baseline associated with higher levels of Aβ in *APOE*4 carriers; red dots). ***D***, Similarly, an interaction between APOE4 genotype and precuneus activation over time (slope) was observed, with a steeper positive slope being associated with higher Aβ burden in *APOE*4 carriers (red dots). Shaded areas refer to the 95% confidence interval.

First, we assessed the predictive effects of baseline precuneus activation and activity change (slope over time derived from LMM) on Aβ- and tau-PET burden (5 years after baseline). With regard to Aβ burden, higher baseline precuneus activity was related to significantly higher whole-brain NAV SUVR (*β* = 0.20; [95% CI 0.05, 0.36]; *t* = 2.544; *p* = 0.012; Fig. 3*A*, Table 4) in the whole sample. Regarding activity change, we observed a significant association between a steeper positive precuneus activity slope and more whole-brain Aβ (*β* = 0.17; [95% CI 0.01, 0.34]; *t* = 2.082; *p* = 0.039; Fig. 3*B*, Table 5). With regard to tau burden, the analysis did not yield a significant effect of baseline precuneus activation or activity slope on entorhinal FTP SUVR (all *p* > 0.05; Extended Data Tables 4-1 and 5-1). Additionally, we note that baseline precuneus activation and the slope of activation over time were positively correlated (*r* = 0.73; [95% CI 0.64, 0.79]; *t*_(149)_ = 12.913; *p* < 0.001).

10.1523/JNEUROSCI.1408-24.2024.t4-1 Table 4-1 Download Table 4-1, DOCX file.

10.1523/JNEUROSCI.1408-24.2024.t5-1 Table 5-1 Download Table 5-1, DOCX file.

**Table 4.** Linear model of effects of activation at baseline on amyloid PET burden

	Whole brain amyloid PET burden							
Predictors	Estimates	Std. error	Std. beta	Standardized std. error	CI	Standardized CI	Statistic	*p*
(Intercept)	0.05	0.28	0.07	0.10	−0.50–0.60	−0.12–0.25	0.18	0.860
Baseline precuneus activity	0.02	0.01	0.20	0.08	0.00–0.03	0.05–0.36	2.54	0.012
Age at baseline	0.00	0.00	0.06	0.08	−0.00–0.00	−0.10–0.22	0.76	0.450
Sex (male)	−0.02	0.02	−0.21	0.18	−0.06–0.01	−0.57–0.15	−1.14	0.256
Education at baseline	−0.00	0.00	−0.13	0.08	−0.01–0.00	−0.29–0.02	−1.74	0.083
Precuneus GMV	0.09	0.31	0.03	0.09	−0.51–0.70	−0.14–0.20	0.31	0.758
Time of baseline MRI to PET	0.00	0.00	0.06	0.08	−0.00–0.00	−0.09–0.22	0.79	0.432
observations	165							
*R*^2^/*R*^2^ adjusted	0.067/0.032							

Box-Cox corrected amyloid PET burden was used as dependent variable, fMRI precuneus activation at baseline, age at baseline, sex, education, precuneus gray matter volume, and time between baseline MRI and PET were used as independent variables. GMV, gray matter volume. CI, 95% confidence interval.

**Table 5.** Linear model of effects of activation over time on amyloid PET burden

	Whole brain amyloid PET burden							
Predictors	Estimates	Std. error	Std. beta	Standardized std. error	CI	Standardized CI	Statistic	*p*
(Intercept)	0.20	0.29	0.03	0.10	−0.37–0.77	−0.17–0.23	0.70	0.487
Precuneus activity slope	1.05	0.51	0.17	0.08	0.05–2.05	0.01–0.34	2.08	0.039
Age at baseline	0.00	0.00	0.01	0.09	−0.00–0.00	−0.16–0.18	0.11	0.916
Sex (male)	−0.01	0.02	−0.10	0.19	−0.05–0.03	−0.48–0.28	−0.51	0.609
Education at baseline	−0.00	0.00	−0.15	0.08	−0.01–0.00	−0.31–0.01	−1.87	0.064
Precuneus GMV	0.02	0.31	0.01	0.09	−0.60–0.64	−0.17–0.19	0.06	0.954
Time of baseline MRI to PET	0.00	0.00	0.06	0.08	−0.00–0.00	−0.11–0.22	0.70	0.486
Observations	151							
*R*^2^/R^2^ adjusted	0.060/0.020							

Box-Cox corrected amyloid PET burden was used as dependent variable, fMRI precuneus activation over time (slope), age at baseline, sex, education, precuneus gray matter volume, and time between baseline MRI and PET were used as independent variables. GMV, gray matter volume. CI, 95% confidence interval.

Second, we assessed the potential moderating effect of *APOE* genotype on the relationship between precuneus baseline activity or activity change and AD burden, thereby including genotype as a group factor in the model. Regarding Aβ burden, the interaction between baseline precuneus activity and *APOE* genotype on whole-brain NAV SUVR was significant (*β* = 0.29; [95% CI 0, 0.57]; *t* = 2.004; *p* = 0.047; Fig. 3*C*, Table 6), such that *APOE*4 carriers with higher baseline activation showed higher future Aβ-PET burden (*β* = 0.33; [95% CI 0.08, 0.58]; *t* = 2.622; *p* = 0.011; Fig. 3*C*, red line). Similarly, there was a significant precuneus activity slope by *APOE* genotype interaction (*β* = 0.39; [95% CI 0.10, 0.69]; *t* = 2.631; *p* = 0.009) on whole-brain NAV SUVR (Fig. 3*D*, Table 7), such that *APOE*4 carriers with a steeper positive activity slope showed higher Aβ-PET burden (*β* = 0.36; [95% CI 0.10, 0.63]; *t* = 2.758; *p* = 0.008; Fig. 3*D*, red line). In contrast, baseline precuneus activation or activity change were not related to future Aβ-PET burden in noncarriers (*p* > 0.05; Fig. 3*C,D*, blue lines). We note that although *APOE*4 carriers had on average higher Aβ-PET burden, the range of SUVR values was similar between groups. With regard to tau burden, we did not observe significant interaction effects between precuneus activity, neither baseline nor slope, and *APOE* genotype on FTP SUVR (all *p* > 0.05; Extended Data Table 6-1, 7-1).

10.1523/JNEUROSCI.1408-24.2024.t6-1 Table 6-1 Download Table 6-1, DOCX file.

10.1523/JNEUROSCI.1408-24.2024.t7-1 Table 7-1 Download Table 7-1, DOCX file.

**Table 6.** Linear model of effects of activation at baseline and *APOE*4 group on amyloid PET burden

	Whole brain amyloid PET burden									
Predictors	Estimates	Std. error	Std. beta	Standardized std. error	CI	Standardized CI	Statistic	Std. statistic	*p*	Std. *p*
(Intercept)	−0.26	0.26	−0.26	0.11	−0.78–0.25	−0.47–−0.05	−1.01	−2.50	0.316	0.014
Baseline precuneus activity	0.00	0.01	0.05	0.10	−0.01–0.02	−0.15–0.25	0.49	0.49	0.622	0.622
*APOE*4 group (carrier)	0.05	0.02	0.80	0.15	0.01–0.09	0.51–1.09	2.37	5.48	0.019	<0.001
Age at baseline	0.00	0.00	0.14	0.08	−0.000.00	−0.01–0.29	1.81	1.81	0.073	0.073
Sex (male)	−0.02	0.02	−0.20	0.17	−0.05–0.01	−0.53–0.13	−1.18	−1.18	0.239	0.239
Education at baseline	−0.00	0.00	−0.09	0.07	−0.01–0.00	−0.23–0.05	−1.31	−1.31	0.191	0.191
Precuneus GMV	0.34	0.28	0.10	0.08	−0.22–0.90	−0.06–0.25	1.20	1.20	0.232	0.232
Time of baseline MRI to PET	0.00	0.00	0.04	0.07	−0.00–0.00	−0.10–0.18	0.58	0.58	0.562	0.562
fMRI Precuneus baseline × *APOE*4 group (carrier)	0.03	0.01	0.29	0.14	0.00–0.05	0.00–0.57	2.00	2.00	0.047	0.047
Observations	165									
*R*^2^/*R*^2^ adjusted	0.235/0.196									

Box-Cox corrected amyloid PET burden was used as dependent variable, fMRI precuneus activation at baseline, *APOE*4 group, age at baseline, sex, education, precuneus gray matter volume, time between baseline MRI and PET, and the interaction of activation at baseline by *APOE*4 group were used as independent variables. GMV, gray matter volume. CI, 95% confidence interval.

**Table 7.** Linear model of effects of activation over time and *APOE*4 group on amyloid PET burden

	Whole brain amyloid PET burden							
Predictors	Estimates	Std. error	Std. beta	standardized std. error	CI	Standardized CI	Statistic	*p*
(Intercept)	−0.16	0.27	−0.30	0.11	−0.69–0.37	−0.51–−0.08	−0.61	0.546
Precuneus activity slope	−0.22	0.64	−0.04	0.10	−1.48–1.03	−0.24–0.17	−0.35	0.727
*APOE*4 group (carrier)	0.08	0.01	0.79	0.15	0.05–0.11	0.49–1.10	5.17	<0.001
Age at baseline	0.00	0.00	0.10	0.08	−0.00–0.00	−0.06–0.26	1.27	0.205
Sex (male)	−0.01	0.02	−0.07	0.17	−0.04–0.03	−0.41–0.27	−0.39	0.695
Education at baseline	−0.00	0.00	−0.11	0.07	−0.01–0.00	−0.26–0.03	−1.54	0.126
Precuneus GMV	0.29	0.29	0.08	0.08	−0.28–0.86	−0.08–0.25	1.00	0.319
Time of baseline MRI to PET	0.00	0.00	0.03	0.08	−0.00–0.00	−0.12–0.18	0.36	0.716
fMRI precuneus slope × *APOE*4 group (carrier)	2.38	0.90	0.39	0.15	0.59–4.16	0.10–0.69	2.63	0.009
Observations	151							
*R*^2^/*R*^2^ adjusted	0.238/0.196							

Box-Cox corrected amyloid PET burden was used as dependent variable, fMRI precuneus activation over time (slope extracted from linear mixed model), *APOE*4 group, age at baseline, sex, education, precuneus gray matter volume, time between baseline MRI and PET, and the interaction of activation over time (slope) by *APOE*4 group were used as independent variables. GMV, gray matter volume. CI, 95% confidence interval.

### Assessment of the relationship between baseline precuneus activation and baseline memory performance as well as changes in memory performance

Partial correlations showed no significant association of baseline precuneus activation with baseline task-fMRI episodic memory performance (rho = 0.13; *p* = 0.09) or with the baseline RBANS delayed memory index score (rho = −0.01; *p* = 0.80). FMRI task performance as measured by the corrected hit rate did not change significantly over time (*β* = −0.06; [95% CI −0.13, −0.01]; *t* = −1.604; *p* = 0.109). However, there was a significant effect of sex (*β* = −0.33; [95% CI −0.58, −0−08]; *t* = −2.597; *p* = 0.010) with male participants showing overall worse performance. No effect of age or education was present (*p* > 0.05). Similar modeling of cognitive performance measured by the RBANS delayed memory index score showed a significant increase over time (*β* = 0.11; [95% CI 0.06, 0.17]; *t* = 3.915; *p* < 0.001). Additionally, we observed a significant effect of sex (*β* = −0.44; [95% CI −0.67, −0.21]; *t* = −3.771; *p* < 0.001) and education (*β* = 0.22; [95% CI 0.11, 0.32]; *t* = 4.143; *p* < 0.001) on longitudinal RBANS delayed memory performance, such that male participants showed lesser improvements in performance over time than female participants and higher education predicted greater performance increases. No effect of age was observed (*p* > 0.05). See Table 8 for an overview over RBANS and fMRI task performance over time.

**Table 8.** Task performance measures over time

	Baseline	3 months	1 year	2 years	4 years
RBANS memory index score	102.11 (9.11); 102	105.36 (9.13); 106	106.23 (7.16); 106	104.78 (8.27); 104	106.18 (9.79); 106
fMRI retrieval task hits	40.65 (5.33);42	40.46 (8.02); 43	37.21 (13.87);43	37.91 (12.25);42	41.02 (5.08);42.5
fMRI retrieval task corrected hit rate	0.73 (0.14); 0.75	0.74 (0.15); 0.75	0.71 (0.17); 0.72	0.71 (0.17); 0.72	0.71 (0.13); 0.73
fMRI retrieval task misses	6.92 (4.59); 6	6.00 (5.26); 4	6.11 (7.05); 4	5.74 (4.68); 5	6.45 (4.18); 5
fMRI retrieval task miss rate	0.14 (0.10); 0.12	0.12 (0.09); 0.08	0.12 (0.10); 0.10	0.12 (0.09); 0.10	0.12 (0.07); 0.10
fMRI retrieval task correct rejections	41.71 (5.01); 43	36.62 (13.80); 41	35.29 (14.18); 41	35.61 (13.26); 40.5	39.35 (7.93); 42
fMRI retrieval task correct rejection rate	0.87 (0.10); 0.90	0.86 (0.11); 0.90	0.83 (0.14); 0.86	0.82 (0.14); 0.88	0.84 (0.12); 0.88

Mean (standard deviation); median for the respective task performance measure per time point of measurement. Corrected hit rate is calculated as hit rate − false alarm rate. Rates are calculated by dividing the number of the respective measure by the number of presented old items (48) for hits and misses or new items (48) for correct rejections. fMRI, functional magnetic resonance imaging; RBANS, Repeatable Battery for the Assessment of Neuropsychological Status.

### Assessment of the effect of baseline precuneus activation and *APOE* genotype on longitudinal memory performance

**Figure 4.**
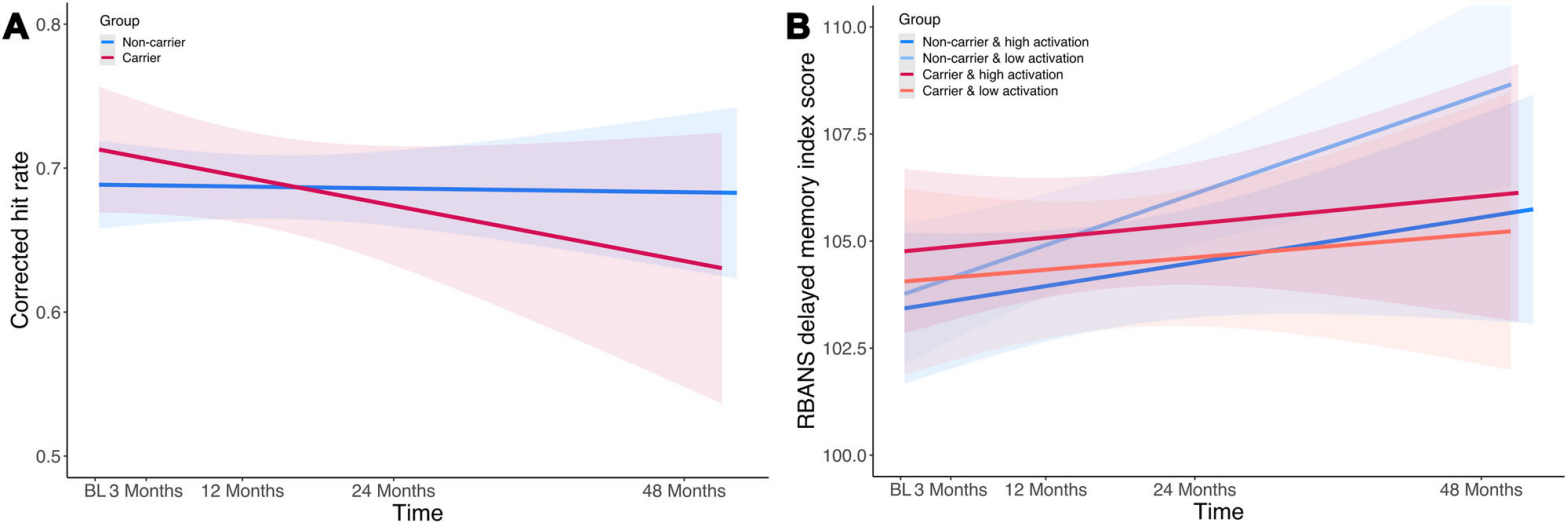
Slope of fMRI task corrected hit rate and RBANS performance. ***A***, Slope of fMRI task corrected hit rate performance over time considering *APOE* genotype. *APOE*4 carriers showed a steeper negative slope (i.e., decline over time) than noncarriers. This suggested that the absence of the *APOE*4 allele is related to a better cognitive outcome (trajectory). ***B***, Slope of RBANS delayed memory performance over time considering *APOE* genotype and precuneus baseline activation. *APOE*4 noncarriers with lower precuneus activation showed a significantly steeper positive slope in RBANS performance over time (corresponding to better delayed memory) in contrast to all other combinations (*APOE*4 noncarriers with high baseline activation and *APOE*4 carriers with low or high baseline activation), suggesting that the absence of the *APOE*4 allele and low precuneus activation at baseline are related to the best cognitive outcomes (trajectory). We split the two *APOE* groups each into a high-and a low-activation group regarding baseline precuneus activation depending on the value being above or below the mean. Shaded areas refer to the 95% confidence interval. BL, baseline. fMRI, functional magnetic resonance imaging; RBANS, Repeatable Battery for the Assessment of Neuropsychological Status.

We assessed whether baseline precuneus activity, *APOE* genotype, or their interaction predicted longitudinal change in memory performance, which showed different results for memory performance for the corrected hit rate in the fMRI task and the RBANS delayed memory index score. In an LMM that included a baseline precuneus activity by session and an *APOE* genotype by session interaction on memory performance, there was a significant *APOE* genotype by session interaction on corrected hit rate (*F*_(4,435)_ = 2.679; *p* = 0.031), but not on RBANS (*p* > 0.05). Regarding the slopes over time for the corrected hit rate, there was a significant effect of *APOE* group ((*β* = −1.51; [95% CI −1.74, −1.27]; *t* = −12.905; *p* < 0.001; Table 9), with *APOE*4 carriers (mean, −0.04; SD = 0.03) showing a steeper negative slope (i.e., decline over time) than noncarriers (mean, −0.00; SD = 0.01), as shown in Figure 4*A*. Post hoc analyses per session revealed that across all sessions, the groups only differed significantly for the 3 month session. *APOE*4 carriers had a higher corrected hit rate compared with noncarriers at the 3 month follow-up session (*t*_(546) _= −2.326; *p* = 0.020; SE = 0.05; [95% CI −0.20, −0.02]). However, in line with the slope results, noncarriers had statistically nonsignificant higher performance than APOE4 carriers at the 24 month (*t*_(499)_ = 0.856; *p* = 0.392; SE = 0.04; [95% CI −0.05, 0.12]) and 48 month (*t*_(582)_ = 1.273; *p* = 0.204; SE = 0.06; [95% CI −0.04, 0.19]) session, as shown in Extended Data Figure 4-1. We note that for the short-term follow-up assessment after 3 months, less than half of the participants had available data for the fMRI task and the RBANS (*N* = 36 *APOE*4 carriers and *N* = 52 for *APOE*4 noncarriers). There was no baseline precuneus activity by session interaction on corrected hit rate or on RBANS (all *p* > 0.05), no significant main effects of *APOE* genotype or baseline activity were found, neither for the corrected hit rate nor for the RBANS (all *p* > 0.05).

10.1523/JNEUROSCI.1408-24.2024.f4-1 Figure 4-1 Download Figure 4-1, TIF file.

**Table 9.** Linear model of effect of *APOE*4 group on changes in corrected hit rate

	fMRI task corrected hit rate slope							
Predictors	Estimates	Std. error	Std. beta	Standardized std. error	CI	Standardized CI	Statistic	*p*
(Intercept)	0.01	0.02	0.60	0.08	−0.04–0.05	0.44–0.76	0.27	0.784
*APOE*4 group (carrier)	−0.04	0.00	−1.51	0.12	−0.05–−0.03	−1.74–−1.27	−12.91	<0.001
Age at baseline	−0.00	0.00	−0.00	0.06	−0.00–0.00	−0.12–0.11	−0.07	0.947
Sex (male)	−0.00	0.00	−0.09	0.12	−0.01–0.00	−0.33–0.15	−0.74	0.458
Education at baseline	−0.00	0.00	−0.05	0.06	−0.00–0.00	−0.16–0.06	−0.89	0.374
Observations	153							
*R*^2^/*R*^2^ adjusted	0.540/0.527							

fMRI task corrected hit rate slope was used as dependent variable, *APOE*4 group, age at baseline, sex, and education, were used as independent variables. CI, 95% confidence interval. fMRI, functional magnetic resonance imaging.

Finally, we tested whether baseline precuneus activity predicted change in memory performance in dependence on *APOE* genotype by extending the previous model by a three-way interaction (activity by *APOE* genotype by session). There was no three-way interaction for corrected hit rate (*p* > 0.05) in the fMRI task. Regarding the RBANS delayed memory index score, the LMM showed a significant baseline precuneus activation by *APOE* genotype by session interaction (*F*_(4,561)_ = 2.5852; *p* = 0.036) on delayed memory performance. We again investigated the performance slopes over time, here for the RBANS, now splitting the two *APOE* groups each into a high- and a low-activation group depending on whether the activity value was above or below the mean. There was a significant effect of the *APOE* activity group ((*β* = −0.28; [95% CI −0.43, −0.13]; *t* = −3.728; *p* < 0.001; Table 10, Fig. 4*B*).

**Table 10.** Linear model of combined effect of *APOE*4 group and baseline precuneus activation on RBANS delayed memory index score

	RBANS delayed memory index slope							
Predictors	Estimates	Std. error	Std. beta	Standardized std. error	CI	Standardized CI	Statistic	*p*
(Intercept)	1.34	1.17	0.14	0.09	−0.97–3.65	−0.04–0.32	1.15	0.253
*APOE* and precuneus activation group	−0.25	0.07	−0.28	0.08	−0.38–−0.12	−0.43–−0.13	−3.73	<0.001
Age at baseline	−0.00	0.02	−0.01	0.08	−0.03–0.03	−0.16–0.15	−0.07	0.941
Sex (male)	−0.43	0.16	−0.44	0.16	−0.75–−0.12	−0.76–−0.12	−2.73	0.007
Education at baseline	0.03	0.02	0.10	0.07	−0.02–0.07	−0.05–0.24	1.29	0.199
Observations	163							
*R*^2^/*R*^2^ adjusted	0.125/0.103							

RBANS delayed memory index score slope was used as dependent variable, *APOE* and precuneus activation group, age at baseline, sex, and education were used as independent variables. We split the two *APOE* groups each into a high- and a low-activation group regarding baseline precuneus activation depending on the value being above or below the mean. CI, 95% confidence interval. RBANS, Repeatable Battery for the Assessment of Neuropsychological Status.

*APOE4* noncarriers (Fig.4*B*, blue lines) with lower precuneus activity showed a significantly steeper positive slope for RBANS over time, corresponding to greater increases in delayed memory performance (*N* = 50; mean, 1.77; SD = 0.21), in contrast to all other APOE and precuneus activation groups (FWE-corrected). This comprised *APOE*4 noncarriers with high baseline activity (*N* = 49; mean, 0.88; SD = 0.32; *t*_(156)_ = 5.202; *p* < 0.001; SE = 0.17) and *APOE*4 carriers (Fig. 4*B*, red lines) with low (*N* = 37; mean, 0.39; SD = 1.58; *t*_(156)_ = 7.776; *p* < 0.001; SE = 0.18) or high baseline activity (*N* = 27; mean, 0.64; SD = 0.67; *t*_(156)_ = 5.428; *p* < 0.001; SE = 0.20). Post hoc analyses on cross-sectional RBANS performance at each follow-up visit between *APOE*4 groups and high and low baseline precuneus activation (fixed at the 25 and 75% percentile; Extended Data Fig. 4-1) revealed that in the group with low baseline activation, there was higher performance for *APOE*4 carriers compared with noncarriers at the 3 month follow up (*t*_(690)_ = −2.124; *p* = 0.034; SE = 2.04; [95% CI −8.33, −0.33]; not corrected for multiple comparisons), but descriptively higher performance for noncarriers at the 24 month (*t*_(507)_ = 1.109; *p* = 0.268; SE = 1.60; [95% CI −1.37, 4.92]) and 48 month (*t*_(544)_ = 1.484; *p* = 0.138; SE = 1.67; [95% CI −0.80, 5.74]) follow-ups. We note again that there was limited data available at the short-term follow-up assessment after 3 months. There were no differences in performance between *APOE* groups with higher activation (all *p* > 0.05; Extended Data Fig. 4-2).

10.1523/JNEUROSCI.1408-24.2024.f4-2 Figure 4-2 Download Figure 4-2, DOCX file.

## Discussion

We utilized PREVENT-AD data to test whether higher precuneus activity at baseline and over time differed by *APOE*4 genotype and whether this was associated with future Aβ or tau burden in cognitively normal older adults. While *APOE*4 carriers did not show higher precuneus activity during retrieval per se, higher baseline activation and change over time were associated with later whole-brain Aβ in this group. We did not, however, observe an effect of precuneus activity on entorhinal tau, suggesting a specific early association between precuneus activity and *APOE*4 genotype for Aβ burden. Finally, our results show a link between brain activity, genotype, and cognition, such that *APOE*4 noncarriers with low precuneus brain activity at baseline show the steepest positive slope over time in an independent delayed memory test.

These results indicate that increased task-based precuneus activation is associated with higher Aβ burden. Previous cross-sectional studies reported associations between higher PMC activation during different cognitive tasks and higher Aβ burden (Sperling et al., 2009; Vannini et al., 2012; Elman et al., 2014; Oh et al., 2015), similar to our longitudinal findings. Interestingly, most studies assessed memory encoding activity (for review, see McDonough et al., 2020; Corriveau-Lecavalier et al., 2024), whereas we investigated increased activity during memory retrieval. Animal models suggest that neuronal hyperexcitability, which may translate to aberrantly higher cerebral activation, facilitates Aβ accumulation (Bero et al., 2011) and is also induced by Aβ-related processes (Zott et al., 2019), therefore potentially forming a vicious cycle. This could suggest that increased blood oxygen level-dependent (BOLD) signal measured in human fMRI studies during memory encoding or retrieval represents neuronal hyperexcitability that is linked to subsequent Aβ accumulation. Interestingly, very early Aβ burden has also been reported in the precuneus (Chételat et al., 2013; Villeneuve et al., 2015; Palmqvist et al., 2017), which is a highly connected and metabolically active hub region of the default-mode network (Buckner et al., 2008). Dynamic causal modeling suggests that increased task activation within PMC regions due to higher Aβ load can drive hyperactivation and tau spread in the medial temporal lobe (MTL; Giorgio et al., 2024), thereby contributing to detrimental processes. This emphasizes the close link between high network activity or connectivity and vulnerability to protein aggregation. While our results support previous findings regarding Aβ, we did not observe associations between fMRI activation in the precuneus and later tau accumulation. Though we did not explicitly assess MTL activity, which might be more closely linked to tau, our results suggest a specific mechanism linking hyperactivation in precuneus with later Aβ.

Our results also show an interaction between precuneus activity and *APOE* genotype. Specifically, we observed that *APOE*4 carriers with higher baseline and longitudinal precuneus activation exhibited higher future Aβ burden. Moreover, higher precuneus activity did not relate to Aβ in the absence of the *APOE*4 allele. While the specific role of *APOE*4 in Aβ accumulation and spread is not fully understood, there is converging evidence for a critical role in various dysfunctional mechanisms that could precipitate AD pathology (Papenberg et al., 2015; Hersi et al., 2017; Najm et al., 2019). For instance, animal models point toward a loss of inhibition in the MTL of *APOE*4 carriers that could drive hyperactivation. However, little is known about the PMC (Nuriel et al., 2017; Najm et al., 2019). Our findings stress the moderating role of the *APOE* genotype on the link between increased activity and Aβ pathology, whereby *APOE*4*-*carrying individuals with increased precuneus activity show higher Aβ accumulation. Moreover, *APOE*4 carriers had higher Aβ burden, thereby replicating previous findings (Chételat and Fouquet, 2013; Liu et al., 2013; Martens et al., 2022). As our *APOE*4 carrier group was primarily composed of heterozygotes with one *APOE*4 allele and only three homozygotes, we did not further distinguish these groups.

Another possibility is increased task-related activity reflecting neuronal or network compensation, which accompanies both normal aging and preclinical AD (Villemagne et al., 2013; Hersi et al., 2017; Salthouse, 2019). Overall, precuneus retrieval activation and behavioral performance decreased over time. Prior cross-sectional studies reported higher task-related precuneus activation in cognitively normal older adults compared with younger adults (Miller et al., 2008; Maillet and Rajah, 2014; Soch et al., 2021). This could, however, be related to higher Aβ accumulation or vascular effects that are often not accounted for in studies on normal aging. Elman and colleagues discussed potential compensatory increases in activation in occipital and parietal areas in Aβ-positive compared with Aβ-negative cognitively normal older adults (Elman et al., 2014). Specific elevated activation might be involved in an attempt at functional compensation to meet task demands (Cabeza et al., 2018; Peelle, 2018) in the presence of early pathological changes and genetic risk. Critically, increased activation could be a compensatory process for a limited time, providing an early advantage that subsequently leads into a vicious cycle of increasing AD pathology and cognitive decline over time (Jones et al., 2017).

Our results show a distinct relationship between baseline activation, *APOE* genotype, and longitudinal episodic memory performance. Specifically, we observed that the steepest positive slope of RBANS performance (i.e., improvement over time) was present in *APOE*4 noncarriers with lower precuneus activity at baseline. While this observation could reflect practice effects (which occur even when using alternating RBANS versions; Calamia et al., 2012) or could be influenced by the relatively high education level in the sample (Samson et al., 2023), this finding suggests that the absence of the *APOE*4 allele combined with lower precuneus activity represents a low-risk profile for cognitive decline. With respect to fMRI recognition performance, we observed a decline in fMRI recognition performance in *APOE*4 carriers over 48 months that was not present in the *APOE*4 noncarriers, with no moderation of cognitive changes by precuneus activity. A stronger decline in recognition memory in cognitively normal *APOE*4 carriers compared with noncarriers has been previously observed (Albert et al., 2014; Morrison et al., 2024). It remains open why our findings differ between different memory measures, with practice effects and moderation by activity for the RBANS memory score but not the fMRI memory task. As such, results should be interpreted with caution, particularly given that data at the 3 month follow-up were only available in approximately half of the sample (Meyer et al., 2019; Tremblay-Mercier et al., 2021). In summary, our results indicate that *APOE*4 carriers show higher risk for memory decline and that this risk might be accentuated in the presence of high precuneus activity.

There are several limitations of our study which should be considered. First, PREVENT-AD is an observational cohort study not initially positioned for the testing of our specific hypotheses. However, given the multifactorial nature of early AD, PREVENT-AD is intentionally designed similar to other large-scale data efforts to allow for the investigation of several hypotheses independently, an approach that is less feasible with traditional study designs. Secondly, fMRI is an indirect measure of neural activity and is influenced by various factors such as the specific task demands and vasculature (Tsvetanov et al., 2021; Corriveau-Lecavalier et al., 2024). Nevertheless, fMRI is a widely validated technique and offers tangible insights into early AD-related brain changes. Third, PET data were only available cross-sectionally and at follow-up with varying interscan intervals. We cannot, therefore, comment on Aβ levels at baseline, nor how pathology changes over time in relation to BOLD. Future analyses could incorporate longitudinal plasma markers of Aβ and tau, as recent data showed faster increase in plasma pTau181 levels over time in *APOE*4 carriers compared with noncarriers in the PREVENT-AD cohort (Yakoub et al., 2023). Fourth, <32% of our included participants were male. While we aimed to account for biological sex, future studies should investigate more balanced samples to avoid biases and inequities associated with unequal sex distribution. Fifth, we did not assess hippocampal activity in our analyses, which could shed further light on the questions at hand. As we observed no significant hippocampal activity related to successful retrieval in our sample; however, we opted to not perform further ROI-based analyses in this region. Future hypothesis-driven inclusion of the hippocampus, in addition to assessing interactions between *APOE*4 and MTL-PMC task and task-independent functional connectivity, could further disentangle the complex relationship between functional features and cognitive performance.

In conclusion, our results suggest that greater precuneus activation during memory retrieval is linked to higher Aβ burden in cognitively normal *APOE*4 carriers. Further, the absence of the *APOE*4 allele in combination with lower precuneus activation could represent a beneficial low-risk profile for future cognitive decline. These findings could advance ongoing research on pharmacological or noninvasive brain stimulation interventions targeting aberrant activity as a therapeutic target for early AD, which is of significant clinical interest in the context of the emergence of the first disease modifying therapies for Aβ accumulation (Budd Haeberlein et al., 2022; Sims et al., 2023; van Dyck et al., 2023). Our study, therefore, represents a timely exploration into the complex dynamics of precuneus activation, *APOE* genotype, Aβ, and cognition in older adults at risk for AD.
