# Supplementary figures and images for: Precuneus Activity during Retrieval Is Positively Associated with Amyloid Burden in Cognitively Normal Older APOE4 Carriers
Source: J Neurosci. 2025 Jan 9;45(6):e1408242024. doi: 10.1523/JNEUROSCI.1408-24.2024 (PMC11800745; doi:10.1523/JNEUROSCI.1408-24.2024)

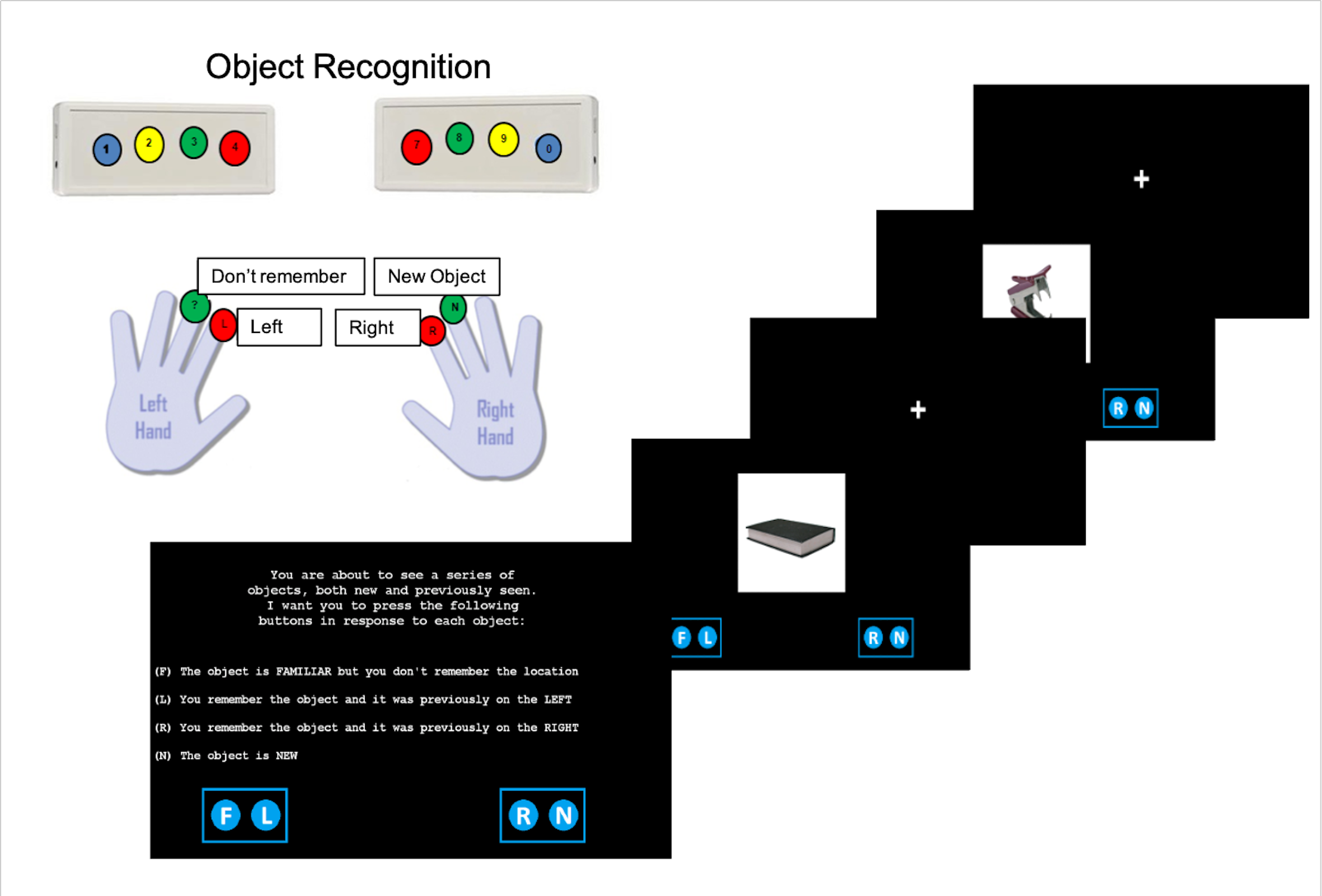

Supplement: Figure 1-1 — Download Figure 1-1, TIF file. [file jneuro-45-e1408242024-s001.tif]

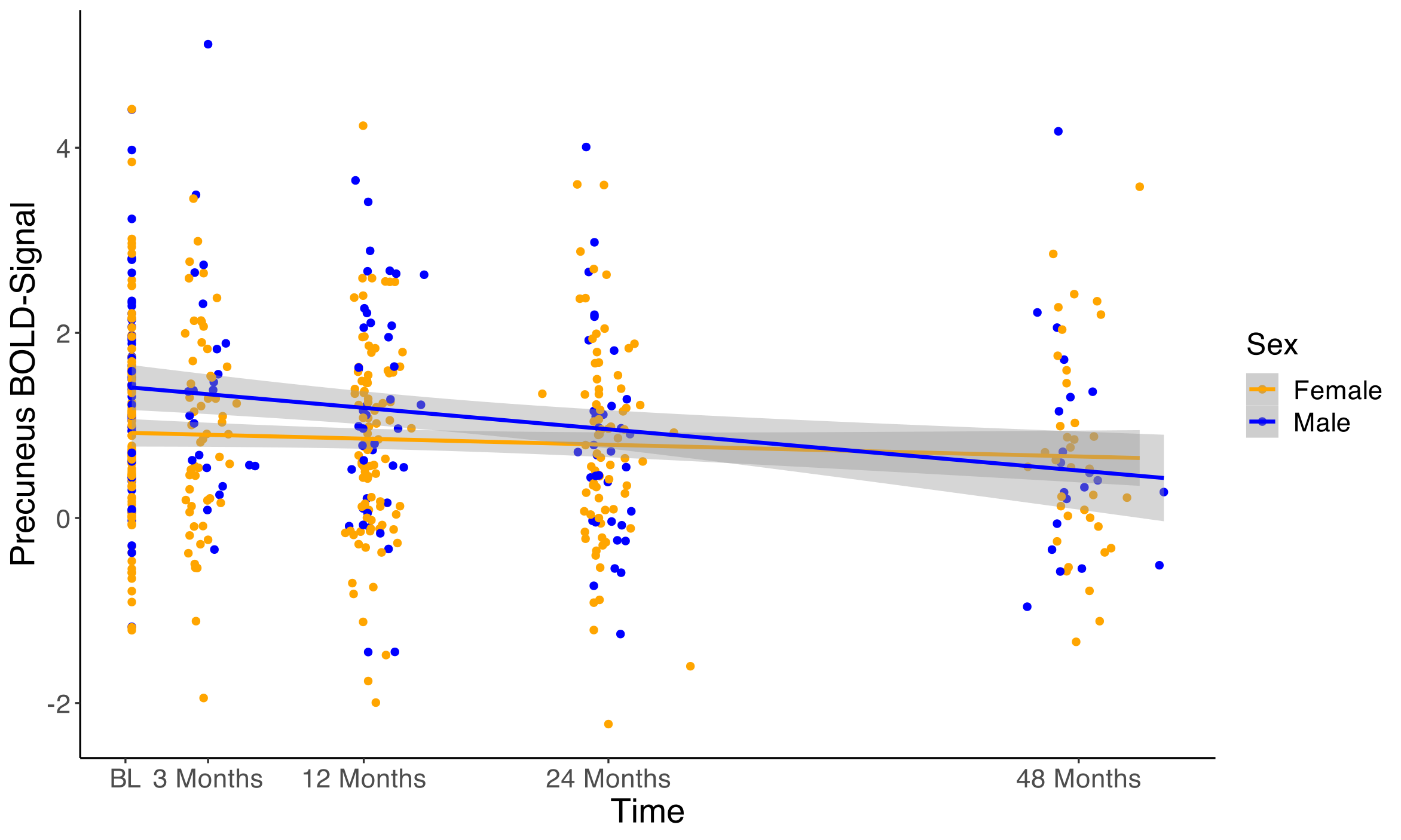

Supplement: Table 3-1 — Download Table 3-1, TIF file. [file jneuro-45-e1408242024-s003.tif]

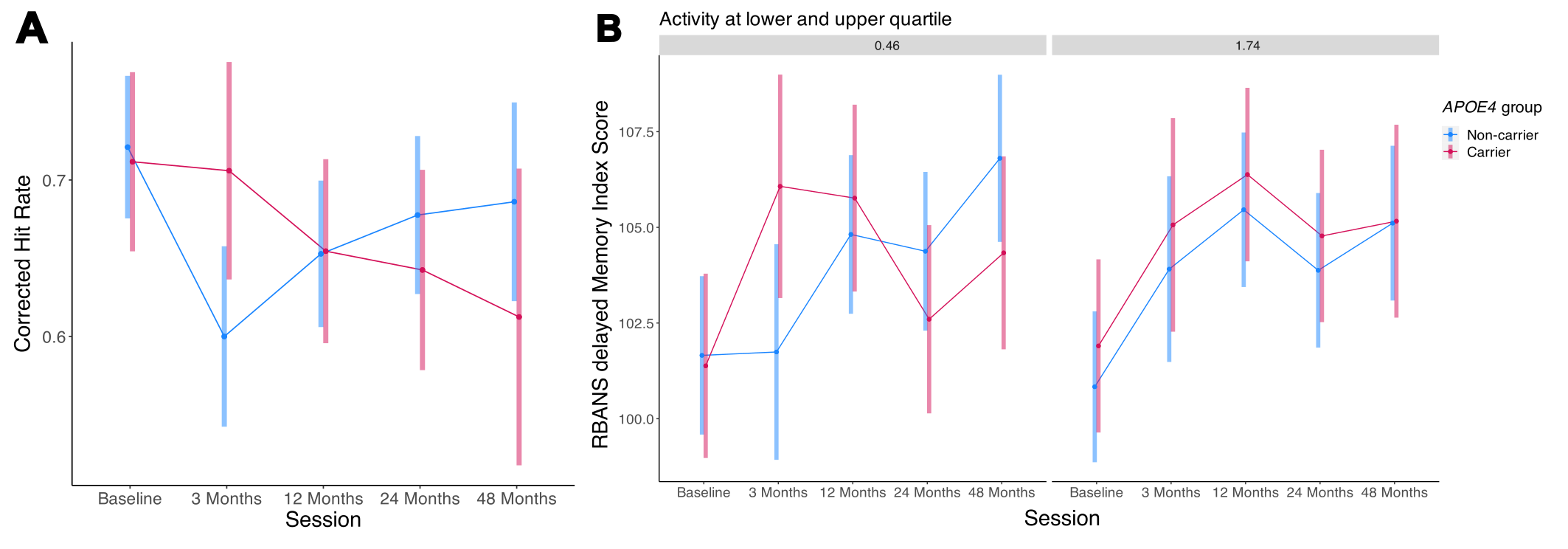

Supplement: Figure 4-1 — Download Figure 4-1, TIF file. [file jneuro-45-e1408242024-s002.tif]
