# Supplementary material for: Precuneus Activity during Retrieval Is Positively Associated with Amyloid Burden in Cognitively Normal Older APOE4 Carriers
Source: J Neurosci. 2025 Jan 9;45(6):e1408242024. doi: 10.1523/JNEUROSCI.1408-24.2024 (PMC11800745; doi:10.1523/JNEUROSCI.1408-24.2024)
Supplement: Table 4-1 — Download Table 4-1, DOCX file. [file jneuro-45-e1408242024-s005.docx]

|  | **Entorhinal tau PET burden** | | | | | | | |
| --- | --- | --- | --- | --- | --- | --- | --- | --- |
| *Predictors* | *Estimates* | *std. Error* | *std. Beta* | *standardized std. Error* | *CI* | *standardized CI* | *Statistic* | *p* |
| (Intercept) | -0.20 | 0.25 | 0.16 | 0.10 | -0.68 – 0.29 | -0.03 – 0.35 | -0.80 | 0.425 |
| Baseline Precuneus Activity | 0.01 | 0.01 | 0.08 | 0.08 | -0.01 – 0.02 | -0.08 – 0.23 | 0.97 | 0.332 |
| Age at Baseline | 0.00 | 0.00 | 0.13 | 0.08 | -0.00 – 0.00 | -0.03 – 0.30 | 1.64 | 0.104 |
| Sex [male] | -0.04 | 0.02 | -0.51 | 0.18 | -0.07 – -0.01 | -0.87 – -0.15 | -2.77 | 0.006 |
| Education Years | 0.00 | 0.00 | 0.03 | 0.08 | -0.00 – 0.00 | -0.12 – 0.19 | 0.43 | 0.670 |
| Precuneus GMV | 0.08 | 0.27 | 0.03 | 0.09 | -0.45 – 0.62 | -0.14 – 0.20 | 0.31 | 0.757 |
| Time Baseline MRI to PET | 0.00 | 0.00 | 0.00 | 0.08 | -0.00 – 0.00 | -0.15 – 0.16 | 0.05 | 0.959 |
| Observations | 165 | | | | | | | |
| R^2^ / R^2^ adjusted | 0.065 / 0.029 | | | | | | | |
