# Supplementary material for: Precuneus Activity during Retrieval Is Positively Associated with Amyloid Burden in Cognitively Normal Older APOE4 Carriers
Source: J Neurosci. 2025 Jan 9;45(6):e1408242024. doi: 10.1523/JNEUROSCI.1408-24.2024 (PMC11800745; doi:10.1523/JNEUROSCI.1408-24.2024)
Supplement: Table 5-1 — Download Table 5-1, DOCX file. [file jneuro-45-e1408242024-s006.docx]

|  | **Entorhinal tau PET burden** | | | | | | | |
| --- | --- | --- | --- | --- | --- | --- | --- | --- |
| *Predictors* | *Estimates* | *std. Error* | *std. Beta* | *standardized std. Error* | *CI* | *standardized CI* | *Statistic* | *p* |
| (Intercept) | -0.10 | 0.25 | 0.13 | 0.10 | -0.59 – 0.38 | -0.07 – 0.32 | -0.43 | 0.671 |
| Precuneus Activity Slope | 0.41 | 0.43 | 0.08 | 0.08 | -0.44 – 1.27 | -0.09 – 0.25 | 0.95 | 0.342 |
| Age at Baseline | 0.00 | 0.00 | 0.12 | 0.09 | -0.00 – 0.00 | -0.06 – 0.29 | 1.33 | 0.187 |
| Sex [male] | -0.03 | 0.02 | -0.40 | 0.19 | -0.06 – -0.00 | -0.78 – -0.02 | -2.09 | 0.038 |
| Education Years | 0.00 | 0.00 | 0.01 | 0.08 | -0.00 – 0.00 | -0.15 – 0.17 | 0.13 | 0.898 |
| Precuneus GMV | 0.03 | 0.27 | 0.01 | 0.09 | -0.50 – 0.56 | -0.17 – 0.19 | 0.10 | 0.917 |
| Time Baseline MRI to PET | -0.00 | 0.00 | -0.03 | 0.08 | -0.00 – 0.00 | -0.20 – 0.13 | -0.41 | 0.682 |
| Observations | 151 | | | | | | | |
| R^2^ / R^2^ adjusted | 0.047 / 0.007 | | | | | | | |
