# Supplementary material for: Precuneus Activity during Retrieval Is Positively Associated with Amyloid Burden in Cognitively Normal Older APOE4 Carriers
Source: J Neurosci. 2025 Jan 9;45(6):e1408242024. doi: 10.1523/JNEUROSCI.1408-24.2024 (PMC11800745; doi:10.1523/JNEUROSCI.1408-24.2024)
Supplement: Table 6-1 — Download Table 6-1, DOCX file. [file jneuro-45-e1408242024-s007.docx]

|  | **Entorhinal tau PET burden** | | | | | | | | | |
| --- | --- | --- | --- | --- | --- | --- | --- | --- | --- | --- |
| *Predictors* | *Estimates* | *std. Error* | *std. Beta* | *standardized std. Error* | *CI* | *standardized CI* | *Statistic* | *std. Statistic* | *p* | *std. p* |
| (Intercept) | -0.31 | 0.25 | 0.02 | 0.11 | -0.80 – 0.18 | -0.20 – 0.25 | -1.25 | 0.20 | 0.214 | 0.841 |
| Baseline Precuneus Activity | -0.00 | 0.01 | -0.03 | 0.11 | -0.02 – 0.02 | -0.24 – 0.19 | -0.24 | -0.24 | 0.812 | 0.812 |
| APOE4 Group [Carrier] | 0.01 | 0.02 | 0.33 | 0.16 | -0.03 – 0.05 | 0.01 – 0.64 | 0.51 | 2.07 | 0.611 | 0.040 |
| Age at Baseline | 0.00 | 0.00 | 0.16 | 0.08 | 0.00 – 0.00 | 0.00 – 0.33 | 1.98 | 1.98 | 0.049 | 0.049 |
| Sex [male] | -0.04 | 0.02 | -0.50 | 0.18 | -0.07 – -0.01 | -0.85 – -0.14 | -2.75 | -2.75 | 0.007 | 0.007 |
| Education Years | 0.00 | 0.00 | 0.06 | 0.08 | -0.00 – 0.01 | -0.10 – 0.21 | 0.73 | 0.73 | 0.467 | 0.467 |
| Precuneus GMV | 0.18 | 0.27 | 0.06 | 0.09 | -0.36 – 0.71 | -0.11 – 0.23 | 0.66 | 0.66 | 0.511 | 0.511 |
| Time Baseline MRI to PET | -0.00 | 0.00 | -0.01 | 0.08 | -0.00 – 0.00 | -0.16 – 0.15 | -0.07 | -0.07 | 0.944 | 0.944 |
| Baseline Precuneus Activity × APOE4 Group [Carrier] | 0.02 | 0.01 | 0.20 | 0.15 | -0.01 – 0.04 | -0.11 – 0.51 | 1.29 | 1.29 | 0.199 | 0.199 |
| Observations | 165 | | | | | | | | | |
| R^2^ / R^2^ adjusted | 0.099 / 0.053 | | | | | | | | | |
