# Supplementary material for: Precuneus Activity during Retrieval Is Positively Associated with Amyloid Burden in Cognitively Normal Older APOE4 Carriers
Source: J Neurosci. 2025 Jan 9;45(6):e1408242024. doi: 10.1523/JNEUROSCI.1408-24.2024 (PMC11800745; doi:10.1523/JNEUROSCI.1408-24.2024)
Supplement: Table 7-1 — Download Table 7-1, DOCX file. [file jneuro-45-e1408242024-s008.docx]

|  | **Entorhinal tau PET burden** | | | | | | | |
| --- | --- | --- | --- | --- | --- | --- | --- | --- |
| *Predictors* | *Estimates* | *std. Error* | *std. Beta* | *standardized std. Error* | *CI* | *standardized CI* | *Statistic* | *p* |
| (Intercept) | -0.18 | 0.26 | -0.03 | 0.12 | -0.68 – 0.33 | -0.27 – 0.21 | -0.68 | 0.497 |
| Precuneus Activity Slope | 0.00 | 0.60 | 0.00 | 0.12 | -1.19 – 1.19 | -0.23 – 0.23 | 0.00 | 0.999 |
| APOE4 Group [Carrier] | 0.01 | 0.02 | 0.18 | 0.20 | -0.02 – 0.05 | -0.21 – 0.57 | 0.90 | 0.370 |
| Amyloid Group [Positive] | 0.01 | 0.05 | 0.11 | 0.56 | -0.08 – 0.10 | -1.00 – 1.23 | 0.20 | 0.842 |
| Age at Baseline | 0.00 | 0.00 | 0.13 | 0.09 | -0.00 – 0.00 | -0.05 – 0.31 | 1.44 | 0.151 |
| Sex [male] | -0.03 | 0.02 | -0.42 | 0.19 | -0.07 – -0.00 | -0.81 – -0.04 | -2.20 | 0.029 |
| Education Years | 0.00 | 0.00 | 0.06 | 0.08 | -0.00 – 0.01 | -0.10 – 0.22 | 0.74 | 0.462 |
| Precuneus GMV | 0.07 | 0.28 | 0.02 | 0.09 | -0.48 – 0.62 | -0.16 – 0.21 | 0.24 | 0.810 |
| Time Baseline MRI to PET | -0.00 | 0.00 | -0.06 | 0.08 | -0.00 – 0.00 | -0.23 – 0.11 | -0.72 | 0.474 |
| Precuneus Activity Slope  × APOE4 Group [Carrier] | 1.16 | 1.07 | 0.22 | 0.21 | -0.95 – 3.27 | -0.18 – 0.63 | 1.09 | 0.279 |
| Precuneus Activity Slope  × Amyloid Group [Positive] | -5.02 | 4.05 | -0.97 | 0.78 | -13.02 – 2.98 | -2.52 – 0.57 | -1.24 | 0.216 |
| APOE4 Group [Carrier] × Amyloid Group [Positive] | 0.03 | 0.05 | 0.32 | 0.62 | -0.07 – 0.13 | -0.91 – 1.56 | 0.52 | 0.603 |
| Precuneus Activity Slope × APOE4 Group [Carrier] ×  Amyloid Group [Positive] | 4.58 | 4.22 | 0.89 | 0.81 | -3.76 – 12.92 | -0.73 – 2.50 | 1.09 | 0.279 |
| Observations | 151 | | | | | | | |
| R^2^ / R^2^ adjusted | 0.119 / 0.042 | | | | | | | |
