# Supplementary material for: Precuneus Activity during Retrieval Is Positively Associated with Amyloid Burden in Cognitively Normal Older APOE4 Carriers
Source: J Neurosci. 2025 Jan 9;45(6):e1408242024. doi: 10.1523/JNEUROSCI.1408-24.2024 (PMC11800745; doi:10.1523/JNEUROSCI.1408-24.2024)
Supplement: Figure 4-2 — Download Figure 4-2, DOCX file. [file jneuro-45-e1408242024-s004.docx]

|  | **RBANS delayed memory index score** | | | | | | |
| --- | --- | --- | --- | --- | --- | --- | --- |
| *Session* | *fMRI baseline activation* | *Estimate* | *std. Error* | *df* | *CI* | *Statistic* | *p* |
| Baseline | Low | 0.28 | 1.58 | 495.70 | -2.83 – 3.38 | 0.17 | 0.863 |
| 3 Months | Low | -4.33 | 2.04 | 689.72 | -8.33 – -0.33 | -2.12 | 0.034 |
| 12 Months | Low | -0.95 | 1.60 | 504.39 | -4.09 – 2.19 | -0.59 | 0.553 |
| 24 Months | Low | 1.78 | 1.60 | 507.18 | -1.37 – 4.92 | 1.11 | 0.268 |
| 48 Months | Low | 2.47 | 1.67 | 543.98 | -0.80 – 5.74 | 1.48 | 0.138 |
| Baseline | High | -1.07 | 1.52 | 501.05 | -4.05 – 1.92 | -0.70 | 0.483 |
| 3 Months | High | -1.16 | 1.88 | 670.54 | -4.85 – 2.53 | -0.62 | 0.539 |
| 12 Months | High | -0.92 | 1.54 | 510.17 | -3.94 – 2.10 | -0.60 | 0.550 |
| 24 Months | High | -0.90 | 1.53 | 507.08 | -3.91 – 2.11 | -0.59 | 0.558 |
| 48 Months | High | -0.05 | 1.64 | 570.74 | -3.27 – 3.17 | -0.03 | 0.975 |
